# Unilateral Facial Paralysis in the Pediatric Patient

**DOI:** 10.7759/cureus.12701

**Published:** 2021-01-14

**Authors:** Kavita Dedhia, Cinzia Marchica, Douglas Mattox

**Affiliations:** 1 Otolaryngology, Children's Hospital of Philadelphia, Philadelphia, USA; 2 Otolaryngology, McGill University, Montreal, CAN; 3 Otolaryngology, Emory University School of Medicine, Atlanta, USA

**Keywords:** arachnoid cyst, facial nerve paralysis, meningioma, sensorineural hearing loss, neurofibromatosis, neurofibromatosis 1, schwannoma, neuroma, hemangioma, internal auditory canal lesion

## Abstract

Unilateral facial paralysis (FP) in the pediatric population is a rare entity secondary to multiple etiologies including infectious, vascular, and neoplastic. In persistent or recurrent FP, imaging can demonstrate a peripheral facial nerve (FN) lesion. Given the rarity of FN lesions, however, there is limited literature regarding optimal management. In this case series, we describe the presentation, evaluation, and management of unilateral FP in three pediatric patients along with a review of the literature. All patients presented with complete FP due to a peripheral FN lesion or compression of the FN. A combined mastoid and middle cranial fossa approach was utilized for excision in two cases, and the other child underwent a translabyrinthine approach. The pathology of the lesions revealed a meningioma, an arachnoid cyst, and a hemangioma. Presentation, evaluation, post-operative outcomes, as well as final pathologies are discussed.

## Introduction

Pediatric facial paralysis (FP) is a rare entity. It affects approximately 5-21/100,000 children per year, with a mean age between five and 11 years and no gender predilection [1]. Common pathologies causing FP are Bell’s palsy, trauma, infections, malignancies, and congenital abnormalities. Variable rates of these diagnoses have been reported in the literature, with Bell’s palsy, infections, and trauma contributing to far more cases of FP than malignancy or chemotherapy [2,3]. Although facial nerve (FN) neoplasms account for only 2% of facial palsies in children, they carry a significantly different prognosis [3]. Diagnosis such as schwanommas, meningiomas, and hemangiomas, and other internal auditory canal (IAC) lesions such as arachnoid cysts and rhabdomyosarcoma must be considered [4]. As a result of the broad differential for FP in children, thorough history taking and physical and diagnostic work-up are necessary for accurate diagnosis and prognosis of FN palsy. Although surgical intervention is infrequently required in these patients, the clinicians should have a heightened awareness of when further diagnostic testing and surgery is warranted.

In this paper, we present three pediatric patients with unilateral FP caused by lesions in the IAC who underwent surgery. The first patient is a 12-year-old male with progressive FN palsy, the second is a three-year-old male with recurrent FN palsy, and the third is a five-year-old male with acute-onset FN palsy. The presentation, diagnosis, and medical as well as surgical management of three pediatric patients with unilateral FP are presented in this case series.

## Case presentation

Case 1

A 12-year-old boy presented to the neurologist with frequent migraine headaches and right-sided progressive FN palsy over three years, with complete paralysis occurring in the last year. Family history was significant for neurofibromatosis type 1 (NF1). Right-sided FP was the only significant finding on physical examination, and no cutaneous manifestations (cafe au lait spots, neurofibromas) of NF1 were visualized. 

**Figure 1:**
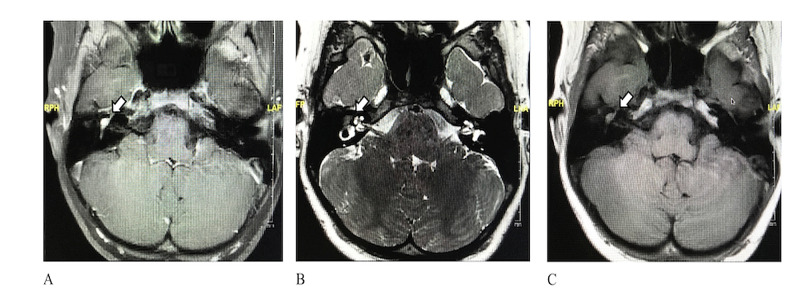
High-resolution MRI of the internal auditory canal in the axial plane. A. T1 post-contrast MRI shows enhancing lesion in the geniculate ganglion. B. T2 MRI shows isointense lesion in the geniculate ganglion. C. T1 pre-contrast MRI shows isointense lesion in the geniculate ganglion.

An MRI revealed a right-sided FN mass involving the geniculate ganglion (GG) and tympanic and labyrinthine segments. The lesion was isointense on T1 and T2 with enhancement on T1 post-contrast (Figure 1). An electromyography/nerve conduction study was indicative of a severe neurogenic lesion affecting the FN at or above the mastoid bone. 

A decision was made to surgically excise the tumor through a combined mastoid and middle cranial fossa approach. At the time of surgery, a 1.5-cm FN neoplasm involving the tympanic and labyrinthine segments was identified. The frozen section of the labyrinthine segment was positive for nerve sheath tumor with calcifications requiring further dissection through the dura for adequate margins. Final pathology depicted positive immunohistochemical staining of the somatostatin receptor 2a, consistent with a final diagnosis of meningioma with World Health Organization (WHO) grade I surgical margins. 

His three-month post-operative visit was notable for subjective improvements in his headaches and dizziness. His post-operative audiogram revealed normal hearing in all the frequencies except for 8,000 Hz, which showed moderate sensorineural hearing loss (SNHL). Subjectively he has no hearing or vestibular concerns. His six-month post-operative MRI does not show recurrence and the one-year post-operative audiogram is stable. 

Case 2

A three-year-old boy presented to his pediatrician with a sudden onset of left facial paresis progressing to FP, over the course of 48 hours. He was diagnosed with Bell’s palsy and started on oral prednisone. Within four months he had complete resolution of the paresis. However, one month later, the left-side palsy recurred. Due to the recurrence an MRI was performed, which demonstrated asymmetric increased contrast enhancement in the left GG. He was treated with steroids and acyclovir with no improvement. He underwent further work-up with genetic testing for NF2 and a repeat MRI. Test was negative for NF2. 

**Figure 2:**
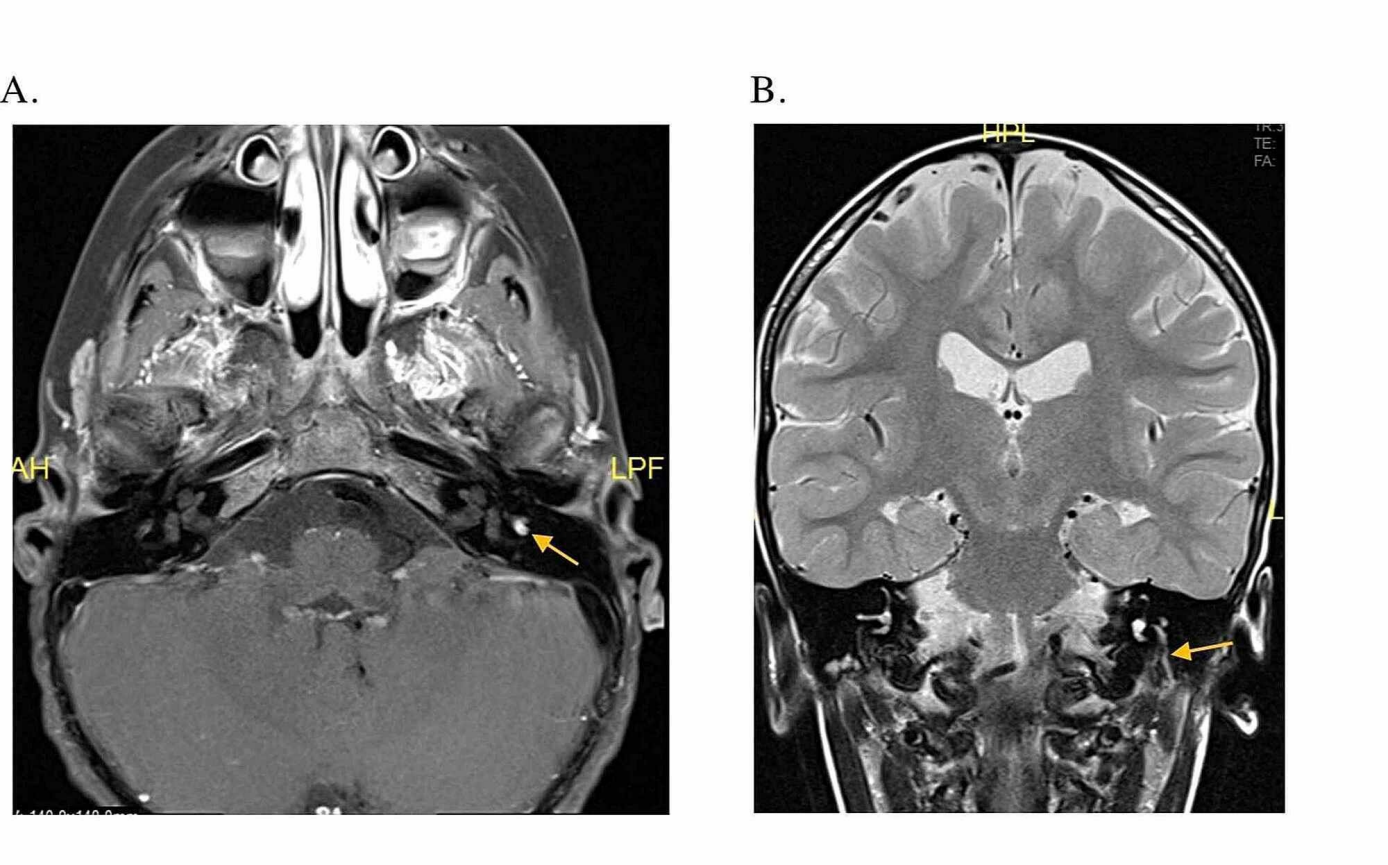
High-resolution MRI of the internal auditory canal. A. Axial T1 post-contrast MRI showing enhancement of the left facial nerve in the geniculate ganglion. B. Coronal T1 post-contrast MRI shows enhancement of the left seventh cranial nerve from the level of the geniculate ganglion all the way down to the stylomastoid foramen. The descending mastoid segment of the facial nerve on the left appears significantly larger than the nerve on the right.

The follow-up MRI showed increased enhancement of the FN as well as a more notable asymmetric enlargement in the mastoid segment (Figure 2). An FN schwannoma was considered in the differential diagnosis, given these findings. The lesion had increased in size with time and there was concern that it may impinge on his cochlear nerve and cause hearing loss. His audiogram was normal, and currently, he did not have any hearing concerns. After discussion with the family, the decision was made to undergo surgical removal of the tumor through a combined transmastoid and middle cranial fossa approach for diagnostic purpose. He also underwent an interposition FN graft with an allograft at the same time. His post-operative course was uneventful and was discharged home on post-operative day (POD) 4. 

At his three-month follow-up, parents did not report any concerns regarding his hearing. Pathology showed hemangioma of the FN. The post-operative audiogram revealed mild-to-moderate hearing loss at 750-1,000 Hz, otherwise unremarkable. 

Case 3

A five-year-old white male presented to the emergency department with one-week history of headache, dizziness, vomiting, and acute onset of FP. Upon presentation, he denied otalgia, tinnitus, or hearing changes. He also denied recent illness, exposure to sick contacts, out-of-country travel, and exposure to ticks.

**Figure 3:**
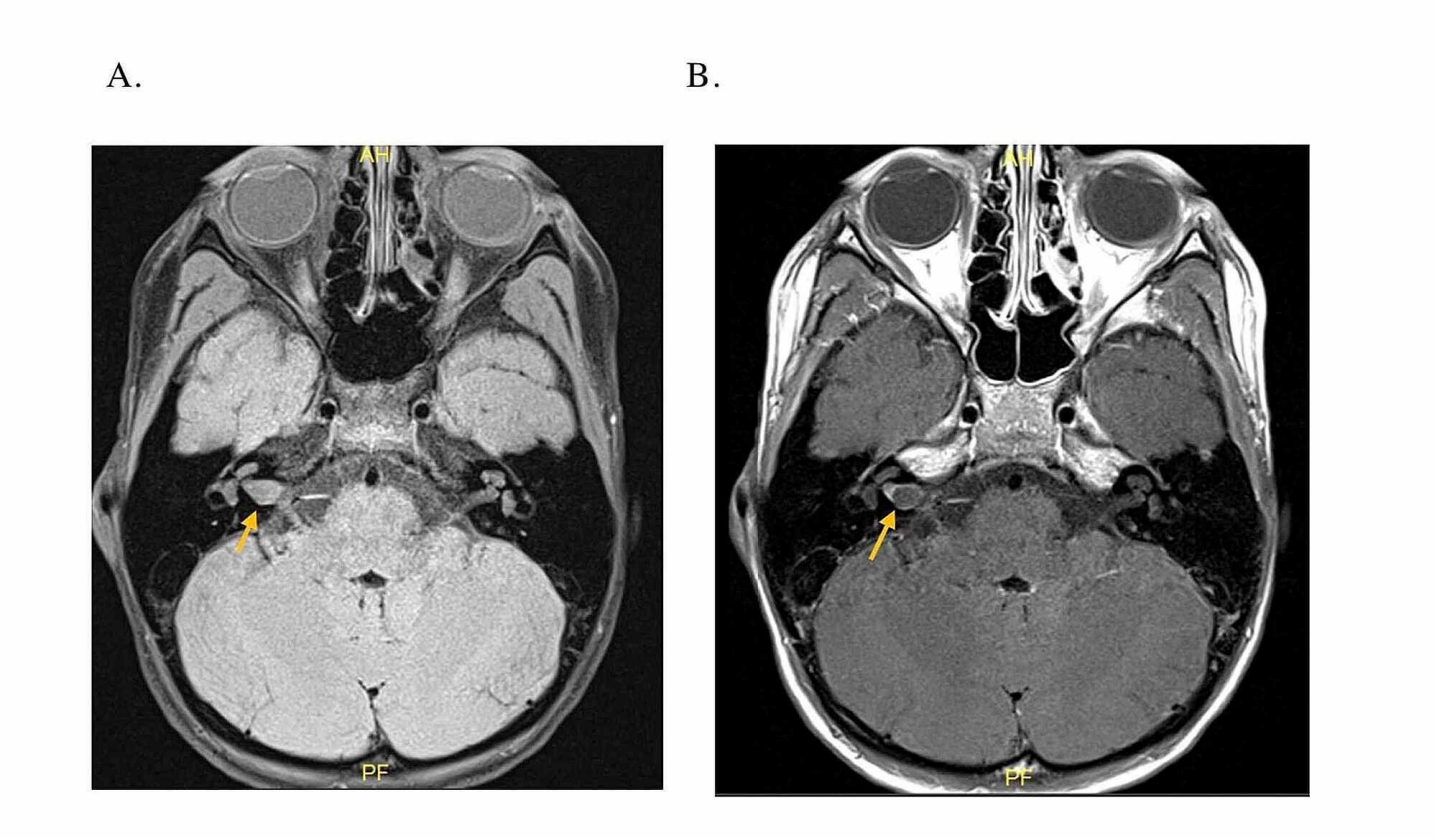
High-resolution MRI of the IAC in the axial plane. A. Demonstrates a T1 sequence without contrast showing mild asymmetric enlargement of the right IAC secondary to a cystic mass. B. T1 post-contrast showing a non-enhancing cystic mass with a fluid level in the right IAC. IAC, internal auditory canal

Examination was normal with the exception of a complete right FP. Initially, a computed tomography (CT) angiogram was negative for stroke, but showed a dilated right IAC. An MRI revealed mild asymmetric enlargement of the right IAC secondary to a small cystic mass measuring 0.7 x 1.0 x 0.6 cm and exerting mass effect over the seventh and eighth cranial nerves (Figure 3). Oral prednisone trial was initiated and the patient was referred to the otolaryngology department. Audiogram work-up revealed mild sloping to severe SNHL with poor word recognition in the right ear, with normal hearing on the left. Based on the history, presentation, and initial imaging, cystic vestibular schwannoma was high on the differential. 

Given the location of the lesion and no serviceable hearing, he underwent a translabyrinthine approach for tumor excision at an outside institution, with the goal of FN preservation. Pathological examination of the specimen revealed an arachnoid cyst. He developed a cerebrospinal fluid (CSF) leak on POD 2, for which he underwent post-auricular exploration and plugging of Eustachian tube with fat graft. At his six-month post-operative visit, his FN function had improved to a House Brackman Grade II/VI, from complete paralysis. The follow-up MRI did not show any cyst re-accumulation. 

## Discussion

Children infrequently present with facial palsy. The most common etiologies are traumatic or an infective process, with tumors accounting for approximately 2%. It is important for these patients to undergo a thorough work-up. This will direct treatment options ranging from antibiotics and steroids for infectious processes, surgical treatment for traumatic or solid malignancies, or even chemotherapy for leukemia patients [5]. 

Accordingly, the work-up for FP in a pediatric patient should include a detailed history and a physical examination to determine the severity and extent of symptoms. Inquisition into trauma, recurrent ear infections, surgical history, travel, radiation exposure, and family history may help tailor diagnostic work-up. Physical examination of the head and neck, with specific attention to the middle ear, can provide a quick and easily treatable diagnosis with consideration to the high rates of traumatic or infectious causes of FP. Further evaluation looking for cutaneous abnormalities associated with NF1, visual and auditory disturbances, and imbalance issues may lend support to an IAC lesion [6]. In cases with an atypical clinical course or presentation, imaging should be done to help identify the etiology. 

In our first case, the patient was diagnosed with FN meningioma. Primary extradural meningiomas represent 1%-2% of all meningiomas, and those located in the middle ear can cause FP [7,8]. A majority are asymptomatic; however, focal cranial nerve deficits (28%), vomiting (53%), and headaches and/or symptoms of increased intracranial pressure (62%) are some reported symptoms in children [8]. Also, there is a higher recurrence rate and a 20%-25% prevalence of atypical to malignant meningiomas in children compared to adults [9,10]. Furthermore, meningiomas occur more frequently in those with a history of radiation exposure or certain genetic disorders (i.e. neurofibromatosis types 1 and 2 and schwannomatosis) [9,11,12]. Observation is reasonable when the mass is asymptomatic and small [13]. Our patient had complete FP with a non-functioning FN and symptomatic headaches; therefore, we decided to proceed with surgical resection of the lesion. Given the location of the lesion, we determined that a combined transmastoid middle fossa approach would be ideal. 

In the second case, the child was initially thought to have Bell's palsy; however, due to the atypical course, imaging was obtained. Imaging initially showed some enhancement in the location of the GG, which progressed to include the mastoid segment of the FN on subsequent MRI. Given the clinical course and MRI findings, we proceeded with surgery. Using this approach, we were able to carefully remove the diseased FN segment and perform an FN graft. The final pathology revealed hemangioma, which rarely involves the GG of the FN, accounting for 0.7% of intratemporal tumors [14,15]. The lesion can be seen to progress distally or proximally to the tympanic or labyrinthine segments of the FN, respectively [16]. Studies have reported accompanying SNHL and cochlear fistula to be present in 25% of cases [14,17]. Imaging often depicts an enlarged FN canal at the location of the hemangioma with the presence of irregular edges. MRI findings may differ based on the age of the hemangioma, but likely to demonstrate increased signal intensity on T2, unevenly distributed, as well as low signals in a point-like distribution [16,18]. Surgical excision with neural preservation is preferred when possible, otherwise grafting should be undertaken. In a series of 18 patients with GG hemangiomas, Semaan et al. [16] demonstrated good results after surgical excision, with 64% hearing preservation. The authors do recommend FN preservation whenever possible due to improved long-term facial function results. However, when this is not possible, we recommend immediate grafting at the time of surgery if possible, for the best FN outcomes. 

The third child with FN paralysis underwent a translabrynthine approach at an outside institution. This is the best approach to remove the lesion and decompress the FN in the setting of no serviceable hearing. The final pathology returned as arachnoid cyst and the patient’s FN recovered to a House Brackman grade II. Arachnoid cysts account for 0.5% of all tumors found in the IAC [4]. Depending on their location and size, the cysts can lead to compression of the nerve trunks to varying degrees as reported in a post-mortem analysis of temporal bones with arachnoid cysts [4]. When present intracanalicular, cysts may also destroy the surrounding cochlear structure, resulting in hearing loss and imbalance [4]. Van la Parra et al. report a case of a six-year old with an arachnoid cyst within the fallopian canal that presented with FP, hearing loss, and CSF otorrhea [19]. CT findings include a low-density, non-calcified mass, which usually demonstrates regular borders that do not enhance with contrast. MRI findings of the arachnoid cyst are similar to the CSF: hypointense on T1 and hyperintense on T2 sequences [20]. These findings are, however, not pathognomonic for a single process; therefore, pre-operative diagnosis is often difficult to ascertain. Given the rarity of these lesions, and the few reported cases in the literature, management is still debated.

## Conclusions

FP is a rare occurrence in the pediatric population and more often warrants inquisition into traumatic or infectious causes though tumors have been recorded to present as FP. In most cases, surgical treatment is not the initial consideration, and children are either observed or managed medically. However, it is important to identify cases that would benefit from surgical resection and understand proper techniques to preserve hearing, balance, and FN function. In cases demonstrating an FN mass or enlargement of the FN canal, rare tumors, such as schwannomas, meningiomas, hemangiomas, and arachnoid cysts, must be considered. Management often requires a multidisciplinary approach with a role for shared decision making between the physicians and the caregivers. Given the potential growth of these lesions, surgical management should be considered with emphasis on preservation of hearing and vestibular function. In select cases, a combined transmastoid and middle cranial fossa approach has led to hearing preservation and the opportunity for FN grafting without complications. These cases highlight the importance of evaluation of neoplastic causes of FP in pediatric patients.
